# Donor age and cell passage affects differentiation potential of murine bone marrow-derived stem cells

**DOI:** 10.1186/1471-2121-9-60

**Published:** 2008-10-28

**Authors:** James D Kretlow, Yu-Qing Jin, Wei Liu, Wen Jie Zhang, Tan-Hui Hong, Guangdong Zhou, L Scott Baggett, Antonios G Mikos, Yilin Cao

**Affiliations:** 1 Department of Bioengineering, Rice University, P.O. Box 1892, MS-142, Houston, TX 77251-1892, USA; 2 Department of Plastic and Reconstructive Surgery, Shanghai 9^th ^People's Hospital, Shanghai Jiao Tong University School of Medicine, Shanghai Key Tissue Engineering Laboratory, Shanghai, 200011, PR China; 3 Department of Statistics, Rice University, P.O. Box 1892, MS-138, Houston, TX 77251-1892,USA; 4 National Tissue Engineering Center of China, Shanghai, 200240, PR China

## Abstract

**Background:**

Bone marrow-derived mesenchymal stem cells (BMSCs) are a widely researched adult stem cell population capable of differentiation into various lineages. Because many promising applications of tissue engineering require cell expansion following harvest and involve the treatment of diseases and conditions found in an aging population, the effect of donor age and *ex vivo *handling must be understood in order to develop clinical techniques and therapeutics based on these cells. Furthermore, there currently exists little understanding as to how these two factors may be influenced by one another.

**Results:**

Differences in the adipogenic, chondrogenic, and osteogenic differentiation capacity of murine MSCs harvested from donor animals of different age and number of passages of these cells were observed. Cells from younger donors adhered to tissue culture polystyrene better and proliferated in greater number than those from older animals. Chondrogenic and osteogenic potential decreased with age for each group, and adipogenic differentiation decreased only in cells from the oldest donors. Significant decreases in differentiation potentials due to passage were observed as well for osteogenesis of BMSCs from the youngest donors and chondrogenesis of the cells from the oldest donors.

**Conclusion:**

Both increasing age and the number of passages have lineage dependent effects on BMSC differentiation potential. Furthermore, there is an obvious interplay between donor age and cell passage that in the future must be accounted for when developing cell-based therapies for clinical use.

## Background

As the prospect of stem cell based therapeutics entering the clinic becomes more of a reality, researchers and clinicians must account for variability among stem cell populations used to evaluate therapeutic modalities in regenerative medicine and also among the patient populations that will potentially provide autogenous or allogeneic stem cells [1-3]. As hinted by the role of stem cell senescence and dysfunction in natural aging [4-7], donor or patient age will be a critical factor that must be accounted for in clinical and laboratory evaluations of stem cell based technology.

There is currently little consensus and in many cases conflicting reports regarding the effect of donor age and cell processing on adult mesenchymal stem cell (MSC) function. A number of studies have previously shown no age related differences in differentiation using human BMSCs [8-11]; however, many studies demonstrating no change in differentiation have found changes in proliferation, attachment, senescence or self-renewal in mouse [12], rat [13,14], and human [15,16] BMSCs. Using mouse adipose derived MSCs (AdMSCs), Shi et al. found an age related decrease in adipogenic differentiation but no difference in osteogenic differentiation [17], while Wall et al. found that with increasing passage, human AdMSCs tended towards osteogenic differentiation over adipogenic differentiation [18]. Similarly, work by Kirkland et al. found that advanced age in rats results in decreased levels of mRNA associated with adipogenic differentiation in preadipocytes [19], a change that has since been linked to decreased expression of CCAAT/enhancer binding protein (C/EBP)-α [20], caused by overexpression of C/EBP homologous protein, and increased release of TNFα [21]. In contrast, studies have found an age related decrease in osteoblastic but not adipogenic differentiation in BMSCs from rats [22] and humans [23,24]. Numerous other studies have found significantly decreased differentiation capability with increasing BMSC donor age, particularly for osteogenic [25-27], chondrogenic [28], and myogenic [29] differentiation.

Another important parameter that must be considered, particularly because of decreased proliferation and the propensity towards senescence observed in cells from aged donors, is the effect of cell passage on the differentiation capability of adult MSCs. BMSCs largely lose their *in vitro *differentiation capability at or around the 6^th ^passage [30,31], but there is evidence of adverse changes as early as the first [32] or second passage [27]. *In vivo *benefits from MSC based therapies are also abated with increased passage [33]. Interestingly, however, while some reports indicate an age related decline in adipogenic differentiation capability for AdMSCs [17] and a similar passage related decline in osteogenic differentiation capability with a simultaneous enhancement in adipogenic differentiation [31], previous results and hypotheses suggested that with increasing passage cells progressed through a lineage hierarchy, whereby bone marrow derived progenitors would retain a capacity towards osteogenic differentiation and adipose derived progenitors towards adipogenicity [34]. Recent comparisons of human BMSC and AdMSC differentiation [35] and transcriptomes [36] supports this hierarchical model of preferential or retained differentiation.

In the only published study that examined the combined effects of increased *in vitro *passages and donor age on BMSC differentiation, Stenderup et al. examined osteogenic and adipogenic differentiation of human BMSCs [16]. They found decreased osteoblastic and adipogenic differentiation with increased number of passages for BMSCs from both young and old donors, but did not observe effects on differentiation when comparing across the two age groups.

To simultaneously evaluate the effects of both age and passage on BMSC differentiation, we utilized a full factorial study design investigating the adipogenic, chondrogenic, and osteogenic differentiation of mouse BMSCs from postnatal, adult, and aged mice at passage 1 and passage 6. The objective of such a study design was to provide a controlled analysis of two variables (age and passage) and possible interaction between these crucial factors in developing adult stem cell based therapeutics and for which no consensus exists regarding their role in MSC differentiation.

## Methods

### Experimental design

This study uses a factorial design to investigate the effects of donor age and cell passage on BMSC differentiation into 3 mesenchymal lineages. Murine bone marrow derived mesenchymal stem cells were harvested from donors aged 6 days (postnatal), 6 weeks (adult), and 1 year (aged) and cultured through either 1 or 6 passages before differentiation was induced. The harvested and cultured cells used were the adherent population of cells within the bone marrow and are often referred to as either marrow stromal cells or mesenchymal stem cells (a sub-population of marrow stromal cells). For this study, the adherent cell population examined will be referred to as bone marrow derived mesenchymal stem cells or BMSCs.

### BMSC harvest and culture

All experiments followed protocols approved by the Animal Experiment and Care Committee of Shanghai Jiao Tong University School of Medicine. Postnatal (6-day old), adult (6-weeks old), and aged (1-year old) transgenic male eGFP C57Bl/6 mice were obtained from a local breeder colony. Mice were euthanized via cervical dislocation and bilateral thoracotomy and then immersed in 75% ethanol for 15 minutes. The bilateral femurs and tibias were aseptically excised, stripped of connective tissues, and the epiphyses and metaphyses were then removed. The remaining diaphyses were placed in a culture dish with 7.5 ml sterile PBS, and the bone marrow was flushed from the shafts via 3–5 minutes of vigorous pipetting of the PBS. The PBS/marrow suspension was then filtered through a 70-μm cell strainer (BD Biosciences, Mississauga, ON, Canada), collected and centrifuged for 5 minutes at 524 × g. After removal of the supernatant, the resulting pellet was resuspended in α-MEM supplemented with 10% fetal bovine serum (FBS, HyClone, Logan, UT), 2 mM L-glutamine and plated at a density of 1.7–2.0 × 10^4 ^cells/cm^2^. Media were changed every 3 days, and cells were passaged when confluent (~5 × 10^4 ^cells/cm^2^) with 0.05% trypsin in 0.02% ethylenediaminetetraacetic acid (Gibco, Canada). Cells were replated at a density of 3 × 10^3 ^cells/cm^2^. First and sixth passage cells were used as indicated for all experiments. Each experiment was performed with cells pooled from 2–7 mice for each age group. Experiments were repeated in triplicate using different batches of marrow isolates.

### Cell attachment and proliferation

After harvesting and resuspension, cells from postnatal, adult, and aged mice were plated individually in wells of 6-well tissue culture plates at a density of 1 × 10^4 ^cells/cm^2^. At 1/2, 1, 2, 4, 8, 16, and 24 hours after plating, media were gently aspirated and the remaining cells were fixed with 4% paraformaldehyde (Sigma, St. Louis, MO) in phosphate buffered saline (PBS) for 15 minutes. Cell adhesion was measured by counting the total cell number per well under fluorescence.

For proliferation, first passage cells from postnatal, adult, and aged mice were plated individually at 2 × 10^3 ^cells/cm^2 ^in wells of 6-well tissue culture plates. At days 1, 2, 4, 6, and 8 cells were trypsinized and counted using a hemacytometer.

Attachment and proliferation assays were performed in triplicate. Cell doublings were calculated as [37]: doublings = log_2 _(number of cells at passage/number of cells seeded).

### Adipogenic differentiation and characterization

As previously described [38], the first or the sixth passage cells were seeded in 2 wells each of a 6-well plate at a density of 3000 cells/cm^2^. Cells were maintained for 24 hours in regular culture media, after which adipogenic differentiation was induced using Dulbecco's modified Eagle medium (DMEM) supplemented with 10% FBS, 0.5 mM isobutylmethylxanthine, 10 μM insulin, 1 μM dexamethasone, and 200 μM indomethacin (all from Sigma) for 3 weeks with full medium changes performed every 3 days. 2 control wells were maintained in DMEM with 10% FBS over the same time course. Each experiment was repeated in triplicate, resulting in 6 total wells for adipogenic differentiation and 6 control wells.

Differentiated and control wells were stained with Oil Red O. At 3 weeks post-induction, wells were gently rinsed with PBS and then fixed with cold 4% paraformaldehyde for 10 minutes. Wells were then washed with 60% isopropyl alcohol (Sigma), incubated for 5 minutes at room temperature in 2% (w/v) Oil Red O reagent (Sigma), and then washed once with isopropyl alcohol followed by repeated washes with distilled water.

For each well, 3 fields were randomly chosen and examined by light and fluorescence microscopy. The number of total cells per field was determined under fluorescence followed by determination of the number of cells containing Oil Red O stained inclusions. A cell containing a visibly stained vacuole was considered to be positively stained. Additionally, for each view the percent area of positive staining was determined using ImageJ (NIH, Bethesda, MD). The area stained was determined by quantifying the actual area stained rather than the area covered by cells containing stained vacuoles. The average percentage was determined from total 3 views of each well and means plus standard deviation were derived from the average percentage of total six wells.

### Chondrogenic differentiation and characterization

As previously described [38], following monolayer culture until the first or the sixth passage, cells were trypsinized, counted, and centrifuged into pelletted micromass cultures (5 × 10^6 ^cells/pellet) in 15 ml conical tubes (BD Biosciences). After centrifugation and culture in regular culture media for 24 hours, chondrogenesis was induced using low glucose DMEM supplemented with 10% FBS, 10 ng/ml TGF-β1, 100 ng/ml IGF (Peprotech, Rocky Hill, NJ), and 10 nM dexamethasone (Sigma). Cells were cultured in induction or control media (low glucose DMEM with 10% FBS only) in incubators with the conical tube lids loosely fastened for 3 weeks and half media were changed every other day. Four pellets (2 for induction, 2 for control) per age/passage batch were cultured, and each batch was repeated in triplicate.

Following culture, 3 pellets from each group were analyzed for type II collagen expression and glycosaminoglycan (GAG) quantification. Immunohistochemical staining was performed as previously described [39]. Briefly, pellets were fixed with 4% paraformaldehyde for 2 h and embedded in Tissue-Tek OCT (Fisher Scientific, Pittsburgh, PA) and sliced into 10 μm thick sections. Samples were blocked and incubated overnight at 4°C in 1:100 diluted mouse anti-Collagen-II (Lab Vision, Fremont, CA) in 1% bovine serum albumin (Sigma) PBS solution. Samples were then incubated with horseradish peroxidase (HRP)-conjugated goat anti-mouse antibody (DAKO, Carpinteria, CA) diluted in phosphate buffered solution (PBS, 1:100) for 30 minutes at room temperature and developed with diaminobenzidine tetrachloride (DAB). Cells were counterstained with hematoxylin. Slides were also stained with Safranin O by being fixed for 10 minutes in 10% formalin in PBS, rinsed with distilled water, and then stained for 2 minutes with 6% Safranin O (Sigma) in distilled water.

Sulfated GAG production was measured from the remaining 3 pellets per group using an Alcian blue binding assay as previously described [40].

### Osteogenic differentiation and characterization

As previously described [38], the first or the sixth passage cells were seeded in 2 wells each of a 6-well plate at a density of 3000 cells/cm^2 ^as similarly performed in 2.3. Cells were maintained for 24 hours in regular culture media, after which they were cultured in low glucose DMEM supplemented with 10% FBS, 0.1 μM dexamethasone, 50 μM ascorbate-2-phosphate, and 10 mM β-glycerophosphate (all from Sigma) to induce osteogenic differentiation. The induction culture was maintained for 3 weeks with full medium changes every 3 days, whereas the control cells were cultured for 3 weeks in low glucose DMEM with 10% FBS. Experiments were performed in triplicate.

Following culture, 3 wells per group were stained with Alizarin Red to visualize calcified deposits. Wells were gently rinsed with PBS and then fixed with 70% ice-cold ethanol for 1 hour. After washing with distilled water, Alizarin Red solution (40 mM Alizarin Red-Tris-HCl, pH 4.1; Sigma) was left at room temperature in wells for 10 minutes. Wells were then extensively washed with distilled water to remove any nonspecific staining, and the stained area was quantified using ImageJ as in 2.4. For the remaining 3 wells per group, calcium cation (Ca^2+^) concentration was determined via a colorimetric assay (Diagnostic Chemicals, Charlottetown, PEI, Canada) as previously described [41].

### Statistical analyses

Analyses of variance (ANOVA) were performed using SAS software (SAS Institute Inc., Cary, NC), followed by Tukey's multiple comparison tests to determine pairwise statistical significance within 95% confidence intervals (*p *< 0.05). All results are reported as means ± standard deviations. Due to quasi-complete separation, binary logistic regression was not performed on the data for collagen II staining of pellets.

## Results

### Postnatal BMSCs exhibit more rapid proliferation and greater attachment than BMSCs from aged donors

**Figure 1:**
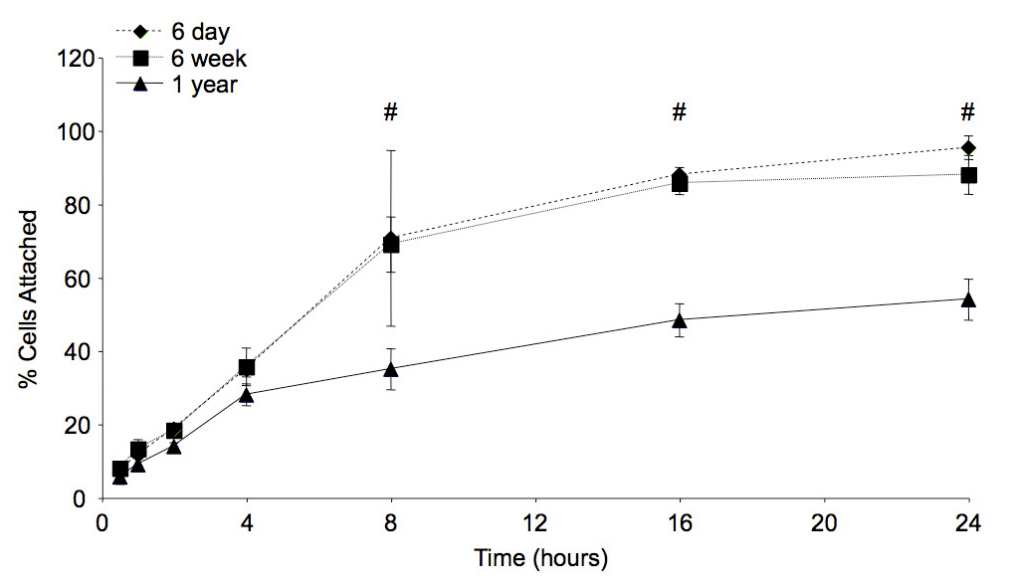
**BMSC Attachment**. Results from the 24-hour assessment of BMSC attachment immediately following harvest. The '#' sign indicates significant differences between cells harvested from both 6-day and 6-week-old mice and those harvested from 1-year-old mice. Error bars designate means ± standard deviation (n = 3, *p *< 0.05).

Following primary harvest, equal numbers of BMSCs were plated in 6-well plates and attachment was measured over 24 hours. Overall, donor age was found to have a statistically significant effect on cell attachment (*p *< 0.0001). As shown in Figure 1, at 8 hours and beyond there were significant differences (*p *< 0.05) in attachment between cells harvested from both 6-day and 6-week-old donors and cells harvested from 1-year-old donors. Over 24 hours there were no significant differences in attachment between cells from 6 day and 6-week-old donors (*p *> 0.05).

**Figure 2:**
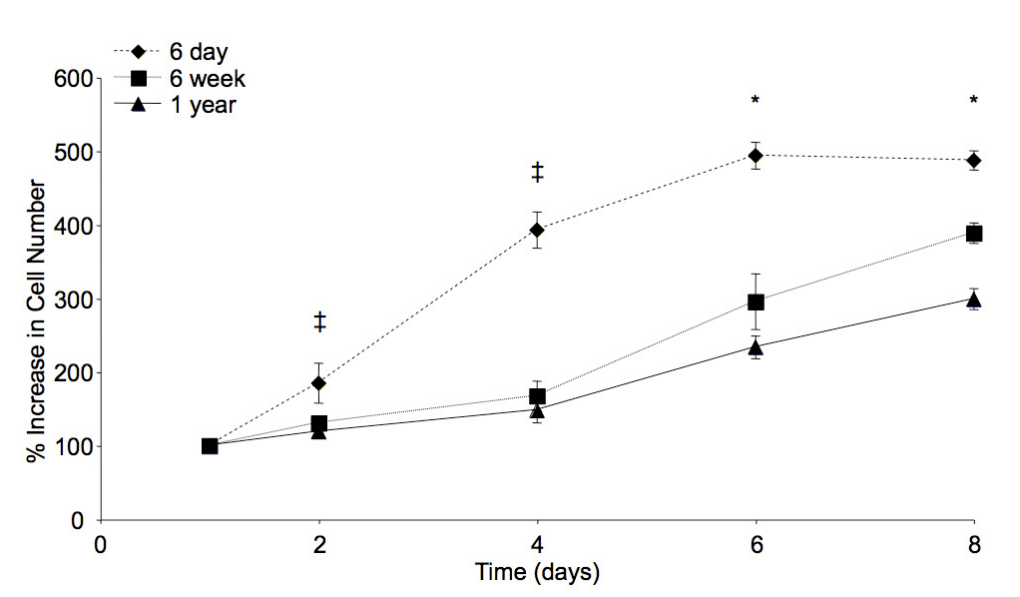
**BMSC Proliferation**. Results from the 8-day assessment of BMSC proliferation during primary cell expansion. The '‡' symbol indicates significant differences between cells harvested from 6-day-old mice and all older donors. The '*' symbol indicates significant differences between groups from all donors. Error bars designate means ± standard deviation (n = 3, *p *< 0.05).

Proliferation was assessed for 8 days following primary harvest. After 2 days there was significantly greater proliferation among cultures harvested from 6-day-old donors compared to cultures from 6-week and 1-year-old donors (*p *< 0.05, Figure 2). This significant difference persisted throughout the 8 days of culture. At 6 and 8 days, significant differences in proliferation were also observed between cultures from 6 week and 1-year-old donors (*p *< 0.05). Overall, donor age was found to have a statistically significant effect on cell proliferation (*p *< 0.0001). There was a 4.9 ± 0.2 fold increase in cell number in cultures of postnatal BMSCs after 8 days compared to cell number after 24 hours, and confluence in these cultures was reached by day 6. An increase of 3.9 ± 0.2 and 3.0 ± 0.1 fold was observed in cultures from adult and aged donors compared to cell numbers after 24 hours, respectively. For the differentiation experiments, passage 1 cells had undergone approximately 2.7 cell doublings, while passage 6 cells had undergone 11.4 doublings. Cells from 6-day-old donors doubled in approximately 2.3 days, with little variation between passage 1 and 6, while cells from 6-week-old and 1-year-old donors had approximate cell doubling times of 3.1 and 3.5 days over the entire culture duration, respectively.

### Adipogenic potential is diminished in BMSCs from aged donors

**Figure 3:**
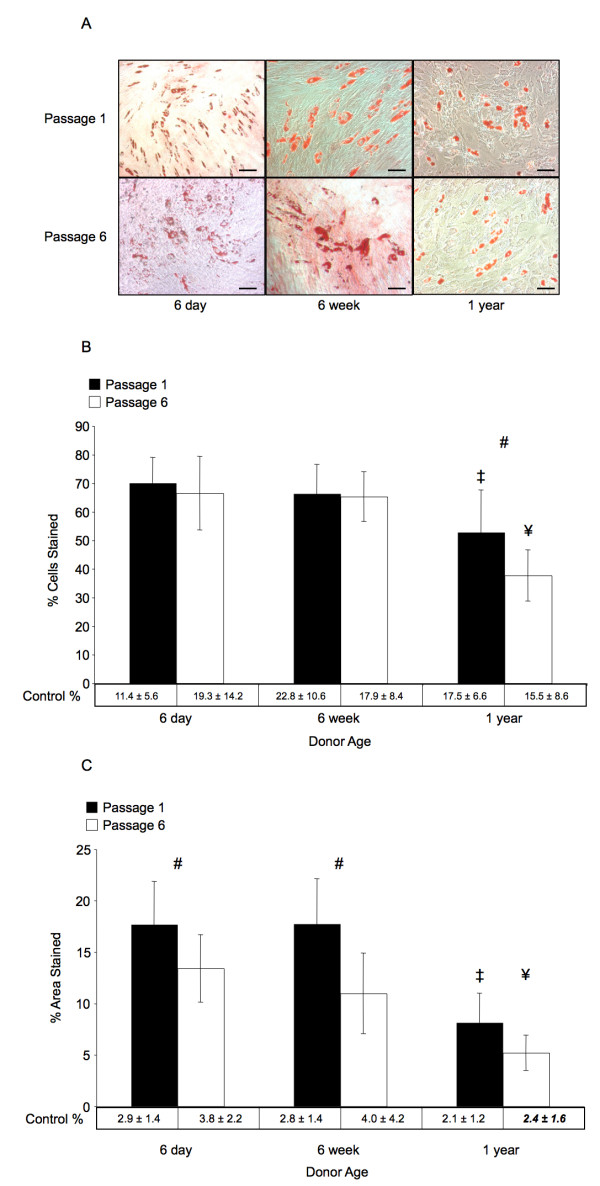
**Adipogenic Differentiation**. Results of adipogenic differentiation after 3 weeks. (*A*) Representative micrographs showing Oil Red O stained lipid inclusions in cultured BMSCs from each experimental group. Magnification bars represent 100 μm in all images. (*B*) Percentage of cells that had intracellular Oil Red O stained inclusions after 3 weeks. Passage related significant difference in staining was only observed in BMSC cultures from 1-year-old donors (*p *< 0.05). (*C*) Percentage of total area that was positively stained with Oil Red O. Passage related significant differences were observed for BMSC cultures from 6-day and 6-week-old donors, and age related differences were only observed between 1-year-old donors and the other groups. The '#' sign indicates a significant difference between passage 1 and passage 6 cultures from similarly aged donors, '‡' indicates a significant difference between the indicated group and any passage 1 groups from younger donors, and '¥' indicates a significant difference between the indicated group and any passage 6 groups from younger donors (*p *> 0.05). Error bars designate means ± standard deviation (n = 6).

To assess the adipogenic potential of BMSCs, Oil Red O staining was quantified following 3 weeks of culture in standard adipogenic media. Quantification was performed in 2 ways, firstly by determining the percentage of cells that contained Oil Red O stained lipid laden vacuoles, and secondly by determining the overall area within a field of view that was stained by Oil Red O. Figure 3 shows results of both quantification methods along with representative histology from each group.

Based on the percentage of cells staining positive for lipid inclusions, there were no significant differences between cells from 6-day and 6-week-old donors, and within these groups there were no differences between cells at passage 1 or 6. A significantly lower percentage of cells stained positive with Oil Red O was found in 1-year-old donors comparing to passage matched numbers of 6-day and 6-week groups (*p *< 0.05). Additionally, within 1-year old group there was also a statistically significant difference between passage 1 and passage 6 cells (52.9 ± 14.7 percent versus 37.7 ± 8.9 percent, *p *< 0.05). Based on the percentage of cells stained, all groups were significantly different from corresponding age and passage matched controls (*p *< 0.05), and there were significant overall effects related to both donor age (*p *< 0.0001) and cell passage (*p *< 0.05). Additionally, there was a significant statistical interaction effect (*p *< 0.05) between donor age and cell passage for this method of quantifying adipogenic differentiation.

Quantifying the area stained with Oil Red O showed no statistically significant differences in adipogenic differentiation of BMSCs between 6-day and 6-week-old donors when compared at the same passage (passage 1: 17.7 ± 4.2 percent versus 17.7 ± 4.4 percent, passage 6: 13.4 ± 3.3 percent versus 11.0 ± 3.9 percent, 6-day and 6-week donors respectively). At the same passage, BMSCs from 1-year-old donors revealed statistically significantly less adipogenic staining area (passage 1: 8.1 ± 2.8 percent, passage 6: 5.2 ± 1.7 percent) than similarly passaged cells from younger donors (*p *< 0.05). For cells from aged donors, there was also no significant effect of increased passages (*p *> 0.05), whereas in the other two donor age groups, passage significantly decreased the area stained (*p *< 0.05). In all groups BMSCs displayed significantly different stained areas than age and passage matched controls (*p *< 0.05) except for passage 6 BMSCs from 1-year-old donors (5.2 ± 1.7 percent vs. 2.3 ± 1.6 percent stained, *p *> 0.05). Significant overall effects were determined for donor age (*p *< 0.0001) and cell passage (*p *< 0.0001); however, there was no statistically significant interaction between donor age and passage based on the area stained (*p *> 0.05).

### Chondrogenic potential decreases with age and repeated passage abrogates chondrogenic differentiation in aged donors

**Figure 4:**
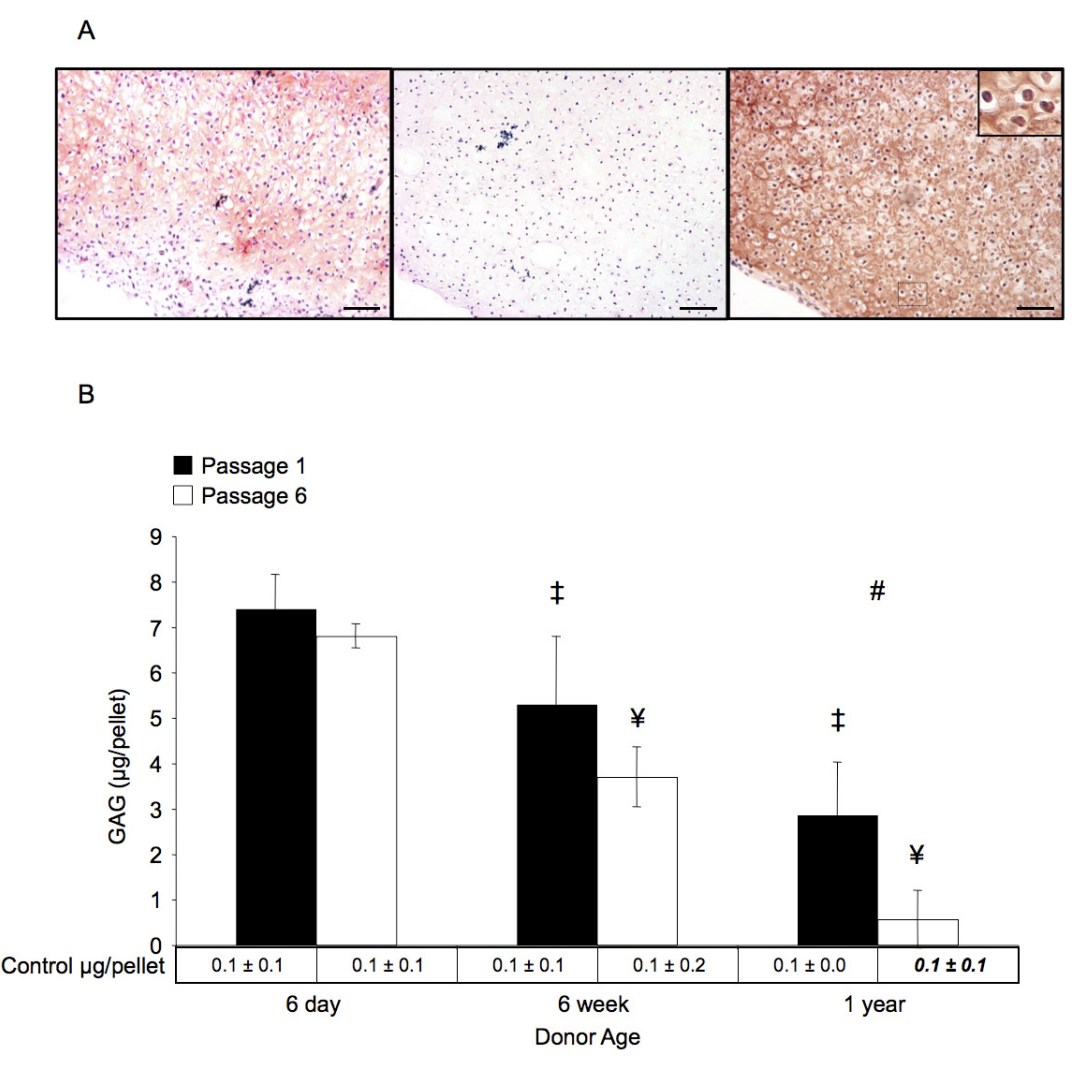
**Chondrogenic Differentiation**. Results of chondrogenic differentiation of pelletted micromasses after 3 weeks. (*A*) Representative micrographs of differentiated micromasses from passage 1 cells of 6-day-old donors. Safranin O (*left*), hematoxylin and eosin (*middle*), and immunohistochemical staining for collagen II (*right*) staining were used to show the morphological structure and biochemical components of induced pellets. The inset demonstrates the lacunar morphology typically observed in cartilage. Magnification bars represent 100 μm in all images. (*B*) Micrograms of sulfated GAG per pellet. The '#' sign indicates a significant difference between passage 1 and passage 6 cultures from similarly aged donors, '‡' indicates a significant difference between the indicated group and any passage 1 groups from younger donors, and '¥' indicates a significant difference between the indicated group and any passage 6 groups from younger donors (*p *< 0.05). At a constant number of passages, significant differences were found across all age groups. Passage related differences were only significant in BMSCs from 1-year-old donors. Error bars designate means ± standard deviation (n = 3).

Sulfated glycosaminoglycan content was quantified for pellets cultured for 3 weeks in chondrogenic media. As shown in Figure 4, compared across donor age groups at constant passage, sulfated GAG content per pellet significantly decreased with each progressive increase in donor age (*p *< 0.05). Increased passage of cells yielded statistically significant differences within donor age matched groups only for BMSCs from 1-year-old donors (2.9 ± 1.2 μg per pellet versus 0.6 ± 0.6 μg per pellet, *p *< 0.05). Additionally, passage 6 BMSCs from 1-year-old donors did not display significantly more sulfated GAG per pellet than passage and donor age matched controls (1-year, passage 6 control: 0.1 ± 0.1 μg per pellet, *p *> 0.05). All other groups had significantly greater GAG content per pellet than passage and age matched controls (*p *< 0.05). Overall, donor animal age (*p *< 0.005) but not cell passage (*p *> 0.1) was found to have a significant affect on the amount of sulfated GAG per pellet.

To further evaluate chondrogenesis, pelleted tissue was immunohistochemically stained for collagen II. As shown in Table 1, with the increase of donor age and cell passage, the number of collagen II-positive pellets decreased. In addition, a typical lacunar structure was observed in histology of collagen II-positive pellets as shown in Figure 4.

**Table 1:** The ratio of collagen II positive pellets to pellets cultured per group, denoted as x:3, where x is the number of pellets staining positively for collagen II

	**6 Day**	**6 Week**	**1 Year**
Passage 1	3/3	3/3	1/3
Passage 6	3/3	2/3	0/3

### Osteogenic potential decreases with age and passage only affects postnatal-harvested BMSCs

**Figure 5:**
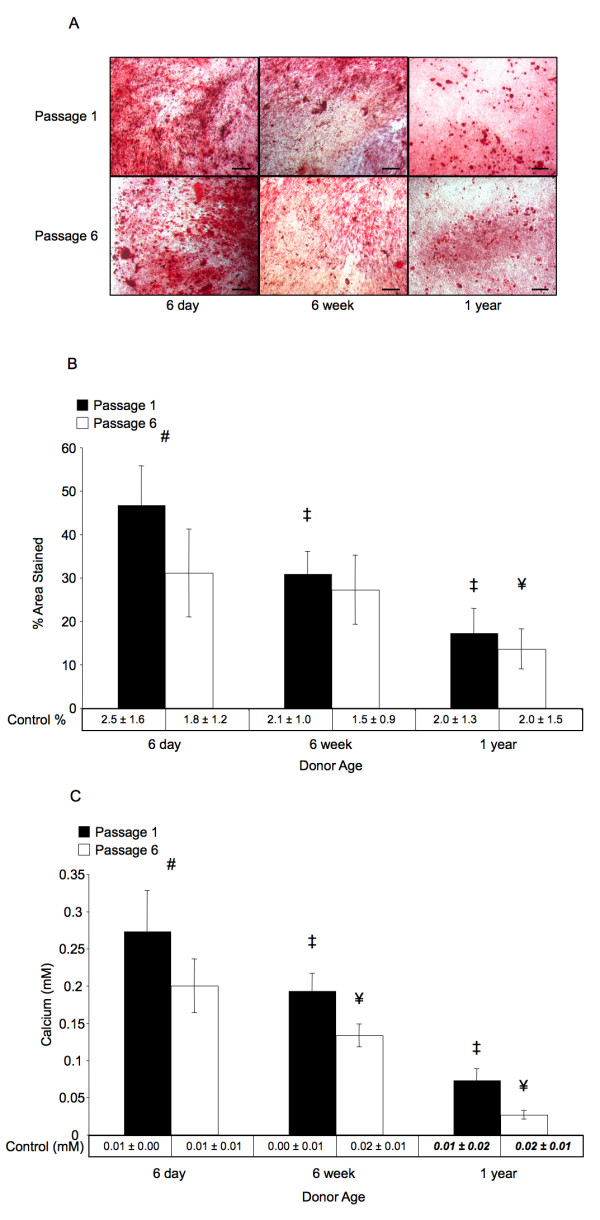
**Osteogenic differentiation**. Results of osteogenic differentiation after 3 weeks. (*A*) Representative micrographs showing Alizarin Red stained mineral deposits on cultured BMSCs from each experimental group. Magnification bars represent 100 μm in all images. (*B*) Percentage of total area viewed that was positively stained with Alizarin Red. (*C*) Quantified calcium cation (mM) determined using a colorimetric calcium assay. For B and C, The '#' sign indicates a significant difference between passage 1 and passage 6 cultures from similarly aged donors, '‡' indicates a significant difference between the indicated group and any passage 1 groups from younger donors, and '¥' indicates a significant difference between the indicated group and any passage 6 groups from younger donors (*p *< 0.05). Age-related decreases in staining and calcium were significant across all age groups, and significant passage related decreases were only observed in BMSCs from 6-day-old donors. Error bars designate means ± standard deviation (n = 3).

Osteogenic potential was determined by quantifying the percent area stained with Alizarin Red, a dye that stains calcium deposition, and by determining the amount of extracellular calcium within a well. Both measures showed that within age groups, the only statistically significant difference based on passage was found in BMSC cultures from postnatal donors (Figure 5, *p *< 0.05), where passage 1 cultures displayed 46.7 ± 9.0 percent area stained and 0.27 ± 0.06 mM Ca^2+ ^versus 31.1 ± 10.1 percent area stained and 0.2 ± 0.04 mM Ca^2+ ^for passage 6 cultures. BMSCs from 6-week-old and 1-year-old donors did not show significant differences in osteogenic potential for cultures at different passages. For both passage 1 and passage 6 cultures, an overall decreased osteogenic potential was found with increased donor age for all ages of donor in terms of both area and calcium content (*p *< 0.05). Tukey's multiple comparison tests revealed a significant difference of any two passage-matched groups comparisons in either stained area or calcium content (*p *< 0.05), except for passage 6 stained area between 6-day-old and 6-week-old donors (31.1 ± 10.1 percent versus 27.2 ± 8.0 percent, *p *> 0.05). Representative micrographs showing Alizarin Red stained samples are also displayed in Figure 5. The percent area stained using Alizarin Red was significantly different from age and passage matched controls (*p *< 0.05); however, the calcium assay showed no significant difference between control and induced groups for both passage 1 and 6 BMSC cultures of 1 year old donors (*p *> 0.05). Analysis of both stained area and calcium content showed a significant effect of donor age (*p *< 0.001); however, analysis of stained area showed a significant overall effect for passage (*p *< 0.001) while analysis of calcium content did not show an overall effect for passage (*p *> 0.05). No statistically significant interaction was found between donor age and passage for either method of evaluation of osteogenic differentiation.

## Discussion

In the present study, we investigated the effects of both donor age and passage on murine BMSC differentiation potentials towards adipogenic, chondrogenic, and osteogenic lineages. Murine BMSCs were used to facilitate future studies performed *in vivo*, where implantation of tissue engineering constructs containing fluorescently labeled progenitor cells allows histologic determination of the cell source for regenerated tissues [42-45]. Additionally, the availability of transgenic mice and mouse cell lines presents the opportunity for tissue engineers to investigate emerging strategies in more clinically appropriate disease models [46]. It is important, however, to note that transgenic animals do not uniformly express GFP, and this expression is variable between tissues with values for murine bone marrow reported at close to 90% [47] in some studies but significantly lower in others [48], thus necessitating a careful comparison of light and fluorescence microscopy when cell numbers are quantified. Routine flow cytometric characterization of the breeder colony used in this study has repeatedly shown over 90% of adherent marrow cells express GFP, even after multiple passages (unpublished data).

No standardized practice exists for the harvest, expansion, and *in vitro *differentiation of BMSCs. As noted in the Materials and Methods section, BMSCs in this study and many others refers to the adherent marrow stromal cells [16,36,49]. Within the adherent BMSC-containing population, our data suggest that a more rapid decline occurs in differentiation potential for osteoblastic and chondrogenic lineages relative to the decline in adipogenic differentiation.

We found that osteogenic and chondrogenic potentials are adversely affected by increased donor age across all three tested donor age groups, while adipogenic differentiation potential is maintained in all but the aged donors (1 year). These results are in agreement with previous work which found that donor age affected osteogenic differentiation of BMSCs more than it affected adipogenic differentiation [24]. Analysis of the transcriptomes of human BMSCs at passage 2 from young donors (average age = 13) suggested that BMSCs should preferentially form bone and cartilage over adipose tissue [36]. Peng et al. recently found similar results, noting that expression of osteogenesis-related genes peaked very early following induction in BMSCs [50]. Other studies addressed the hypothesis that age related decreases in bone regeneration were due to BMSC aging, resulting in a decreased osteogenic potential with a concurrent increase in adipogenic potential [8]. In this study BMSCs maintained their potential for adipogenic differentiation in early aging but exhibited decreased potential for chondrogenic and osteogenic differentiation, and, although no absolute increase in adipogenic potential was observed with increasing age, the relative differences between differentiation potentials with age would thus favor adipogenesis over osteogenesis and chondrogenesis. Age related cellular dysfunction has been hypothesized to be the cause of multiple diseases of bone and cartilage associated almost exclusively with aging including osteoarthritis and osteoporosis, and loss of progenitor cell differentiation potential could contribute to these diseases [51]. The present study supports these hypotheses.

The effect of *in vitro *culture period prior to the induction of differentiation was also investigated by expanding cells through 6 passages and comparing differentiation to cells after a single passage. For chondrogenesis, increased passage only affected cells from 1-year-old donors, rendering them equal to control groups at the level of significance (*p *> 0.05). For osteogenesis, the opposite effect was observed; a difference in osteogenesis due to passage alone was only observed in BMSC cultures from postnatal mice. Previous work using human BMSCs observed a greater passage related decrease in osteogenic differentiation in cells from young donors compared to cells from aged donors [16]. Early passage postnatal BMSCs may be preferentially inclined towards osteogenesis and this preference may be quickly eliminated with repeated passages and aging, leading to the observed differences between passage 1 postnatal BMSCs and all other groups with respect to osteogenic differentiation.

The effect of passage on adipogenesis was more obscure. Quantifying the percentage of cells stained with Oil Red O showed significant passage related differences only in cultures from aged donors, whereas quantifying the percentage area stained showed significant differences in cultures from postnatal and adult donors but not those from aged mice. A previous study investigating adipogenic differentiation with increasing BMSC passages found that the size of adipocytes decreased with increased passage [16]. Thus for postnatal and adult derived BMSCs, passage related changes in area might be largely due to decreased cell volume rather than a decrease in the number of differentiated cells. In cultures of BMSCs from aged donors, the relatively decreased number of cells undergoing adipogenic differentiation may render this effect statistically insignificant at the designated sample size (n = 6). When characterizing adipogenic differentiation via quantification of the percentage of cells stained with Oil Red O, the statistically significant interaction effect of donor age and passage reflects that cultures from aged donors were the only group to be both significantly decreased from other age groups and to have a significant decrease in cells stained between passage 1 and passage 6 cultures. This may reflect a loss of adipogenic differentiation capacity only experienced by MSCs from aged donors that is not detected when quantifying the stained area due to passage-related decreases in adipocyte size experienced in cultures from all donors. It is also important to note that the selected adipogenic cocktail utilized indomethacin, a commonly used chemical for these applications that inhibits cyclooxygenase. Indomethacin has been shown to both positively and negatively effect PPARγ in a concentration dependent manner [52]. Although adipogenic differentiation can be induced via PPARγ dependent and independent signaling [53], PPARγ 2 activation may be critical in BMSC differentiation as this pathway promotes terminal differentiation and suppresses *Osf2/Cbfa1 *[54]. A PPARγ ligand such as rosiglitazone may therefore be a more ideal component for adipogenic induction media for BMSCs.

Working with human BMSCs, Banfi et al. found decreased adipogenic, chondrogenic, and osteogenic potentials when increasing from passage 1 to passage 5 and found adipogenic potential to be compromised prior to osteo- or chondrogenic potential [32]. In the present study, passage effects were variable when considered along with specific donor ages. For example, for 6-week-old donors, osteogenesis and chondrogenesis were unaffected by passage, but adipogenesis as measured by percent area stained was significantly decreased with increased passage. These results correlate well to published studies using human BMSCs [32,34]; however, it should be noted that due to the use of biochemical characterization methods in addition to histology to characterize osteogenic and chondrogenic differentiation, the sample size for these methods was smaller (n = 3) than for evaluation of adipogenic differentiation (n = 6). Passage adversely affected osteogenic potential in BMSCs from postnatal donors, while chondrogenesis was only diminished by passage in BMSCs from 1-year-old donors, suggesting differences in passage effects for different differentiation lineages at different ages. When evaluating changes associated with passage, it is important to note that the adherent marrow stroma is a heterogeneous cell population, therefore there is concern that changes attributed to altered MSC function could actually be due to the preferential proliferation of one or several type(s) of cell(s) over others. The observed variable effects of passage with age suggest that different or additional factors other than BMSC number and differences in attachment/proliferation contribute to differences in differentiation potential, as, in the case that heterogeneity and subsequent differential proliferation, one would expect effects due to frequency or proliferation to be exhibited across all lineages.

## Conclusion

Based on the results of this study and many other previous studies, it appears that many variables must be considered when choosing an ideal or appropriate cell source for a specific application. Future studies should address tissue regeneration *in vivo*, as this will allow parameters such as aging and passage to be compared using criteria more closely related to the clinical goal of regenerating destroyed or dysfunctional tissues. As more is learned regarding aging and passage of adult stem cells, it may be determined that a certain population of cells is more appropriate for specific therapies based on patient age and the tissue of interest. While the present study offers little in the way of mechanistic explanations for the observed phenomena, it provides one of the first analyses of age in combination with passage in an animal model that will be useful for developing future tissue engineering strategies. There is little doubt that aging of adult stem cells plays a role in the spectrum of changes during normal aging and that consideration of age and passage in combination will prove to be critical to the success of any strategy that seeks to regenerate tissue through the use of implanted progenitor cells.

## Authors' contributions

JDK participated in the conception of the study, experimental design, execution, data collection and analysis, and drafted the manuscript. YQJ participated in the conception of the study, experimental design, execution, data collection, table and figure design, and revised the manuscript. WL conceived of the study and participated in its design, coordination, data analysis, and made significant contributions to the drafting and editing of the manuscript. WJZ assisted with the conception of the study and its design and coordination. HTH assisted with the execution of the MSC harvests, cell culture, and data collection. GZ assisted with the conception of the study and immunohistochemistry data collection. LSB performed the statistical analyses and assisted in drafting the manuscript. AGM participated in the conception of the study, data analysis, and both drafting and editing the manuscript. YC conceived of the study and assisted with the coordination of the experiments and editing the manuscript. All authors read and approved the final manuscript.
